# Around the Hospitals

**Published:** 1894-03-17

**Authors:** 


### AROUND THE HOSPITALS.

Notice to the Managers of Institutions.—Special space is now reserved for the insertion of notices of hospital meetings and festivals. The Editor requests that all notices may reach The Hospital Office, 428, Strand, at least one week before the date of each meeting.

**Southport Infirmary.**—This is one of those more fortunate hospitals which have been able to close the year with a balance in its favour. The balance is small but creditable, since the patients in the wards out-numbered those of the previous year by 62. The new infirmary is progressing; the contract to the builders is £16,409. Besides other expenses, £3,000 is needed for furnishing. £18,536 has been subscribed to the present time.

**The Kent and Canterbury Hospital.** — The amount of enthusiasm evoked on the occasion of the collection to celebrate the centenary of the institution was certainly not great, the subscriptions being ridiculously small. Further, the receipts from ordinary sources showed a diminution during the past year, and no legacies were received. Such is not the encouragement which the hospital should receive, as will be readily admitted when the careful administration of the funds is considered, whilst the in-patients were only reduced by twelve; the housekeeping, stationery, and house expenses show a decided diminution. In 1891 the total ordinary expenditure amounted to £4,065; in 1893 it only equalled £3,555. Whilst the management of the institution seems inclined to a policy not universally adopted of limiting expenditure to income, it would be indeed a reproach to Canterbury were the work of healing curtailed for want of necessary funds. During 1893 an outbreak of scarlet fever has brought home the need of an isolation ward. This of course every general hospital should possess. The result of no such means of separating infectious cases from the wards has been that the disease spread amongst a certain number of patients, and one of the male wards had to be requisitioned for the reception of the cases. At the annual meeting of governors it was stated that the city would have to provide a fever hospital, and in that case would receive any cases occurring in the hospital. It was therefore decided that additions of another nature, namely, new quarters for the nurses, should be carried out in the first instance, the need for improved accommodation being most pressing. During the past year the drainage of the hospital has been much improved. It was necessary to encroach on the capital to provide the necessary funds.

**The Ulster Hospital for Women and Children.**—This institution has now attained its majority, and ends the year in a sound financial condition. The in-patients numbered 272, and the out-patients 8,541, both classes showing an increase over the numbers of the previous year. Whilst several good friends of the hospital have been removed by death, a large number yet remain who show their interest in a substantial manner. A bazaar in aid of the funds of the institution realised £330, a sum only to be obtained, now that bazaars are somewhat overdone, by great energy and interest on the part of the promoters. At the annual meeting of the institution tribute was paid to Miss Stewart and her nurses. The accounts of the hospital show a balance of £127 to the good.

